# Supplementary material for: Deletion of wfs1 Impairs Oligodendrocyte Precursor Cells Dorsal Distribution and Myelination Through the wfs1-hmgcs1 Axis in Zebrafish
Source: Biology (Basel). 2026 Aug 16;15(16):1402. doi: 10.3390/biology15161402 (PMC13510151; doi:10.3390/biology15161402)
Supplement: Supplementary file 1 [file biology-15-01402-s001.zip › File S1-Transcriptome sequencing data and differentially expressed genes (DEGs) in wfs1-- zebrafish at 5 dpf/2.KEGG/wfs1KOvsWT/GSEA/WT/PROTEIN_EXPORT/PROTEIN_EXPORT(DRE03060).html]

Details for gene set PROTEIN\_EXPORT(DRE03060)[GSEA]

|  || Dataset | deg.sample |
| Phenotype | sample.cls |
| Upregulated in class | WT |
| GeneSet | PROTEIN\_EXPORT(DRE03060) |
| Enrichment Score (ES) | -0.32226017 |
| Normalized Enrichment Score (NES) | -1.2898369 |
| Nominal p-value | 0.17819707 |
| FDR q-value | 0.5864564 |
| FWER p-Value | 0.987 |
Table: GSEA Results Summary

  

Fig 1: Enrichment plot: PROTEIN\_EXPORT(DRE03060)      
 Profile of the Running ES Score & Positions of GeneSet Members on the Rank Ordered List

  

| PROBE | DESCRIPTION (from dataset) | GENE SYMBOL | GENE\_TITLE | RANK IN GENE LIST | RANK METRIC SCORE | RUNNING ES | CORE ENRICHMENT || 1 | 436794 | sec11a |  |  | 633 | 2.715 | 0.1471 | No |
| 2 | 445299\_1 | immp2l |  |  | 1733 | 1.515 | 0.2058 | No |
| 3 | 436845 | srprb |  |  | 5930 | 0.594 | 0.1106 | No |
| 4 | 436861 | sec63 |  |  | 6659 | 0.523 | 0.1200 | No |
| 5 | 378848 | hspa5 |  |  | 8207 | 0.415 | 0.0969 | No |
| 6 | 266796 | sec61a1l |  |  | 9478 | 0.335 | 0.0777 | No |
| 7 | 446119 | srp68 |  |  | 10323 | 0.283 | 0.0686 | No |
| 8 | 192298 | sec61a1 |  |  | 13031 | 0.138 | -0.0078 | No |
| 9 | 393963 | srp54 |  |  | 13988 | 0.093 | -0.0321 | No |
| 10 | 795154 | immp1l |  |  | 17602 | -0.058 | -0.1419 | No |
| 11 | 405884 | spcs3 |  |  | 18297 | -0.089 | -0.1582 | No |
| 12 | 563660 | sec62 |  |  | 18670 | -0.107 | -0.1633 | No |
| 13 | 541342 | spcs2 |  |  | 20221 | -0.176 | -0.2011 | No |
| 14 | 394071 | srp9 |  |  | 21127 | -0.222 | -0.2158 | No |
| 15 | 327335 | srp72 |  |  | 21356 | -0.234 | -0.2086 | No |
| 16 | 569063 | srpr |  |  | 21485 | -0.240 | -0.1978 | No |
| 17 | 436794\_1 | sec11a |  |  | 21578 | -0.245 | -0.1857 | No |
| 18 | 791986 | sec61b |  |  | 21981 | -0.266 | -0.1819 | No |
| 19 | 406652 | srp19 |  |  | 22993 | -0.327 | -0.1935 | No |
| 20 | 606666 | spcs1 |  |  | 23971 | -0.390 | -0.2002 | No |
| 21 | 619260 | srp14 |  |  | 24740 | -0.433 | -0.1977 | No |
| 22 | 436772\_1 | sec61g |  |  | 25191 | -0.459 | -0.1836 | No |
| 23 | 562963 | oxa1l |  |  | 27871 | -0.725 | -0.2231 | Yes |
| 24 | 436794\_2 | sec11a |  |  | 31031 | -1.999 | -0.1993 | Yes |
| 25 | 445299 | immp2l |  |  | 31698 | -3.680 | 0.0060 | Yes |
Table: GSEA details [plain text format]

  

Fig 2: PROTEIN\_EXPORT(DRE03060)      
 Blue-Pink O' Gram in the Space of the Analyzed GeneSet

  

Fig 3: PROTEIN\_EXPORT(DRE03060): Random ES distribution      
 Gene set null distribution of ES for **PROTEIN\_EXPORT(DRE03060)**
